# IELTS Band 8 Strategies for Busy Physicians From Non-native English-Speaking Countries

**DOI:** 10.7759/cureus.36838

**Published:** 2023-03-29

**Authors:** Rajmohan Seetharaman

**Affiliations:** 1 Pharmacology and Therapeutics, Seth G.S. Medical College and King Edward Memorial (KEM) Hospital, Mumbai, IND

**Keywords:** challenges faced, preparation strategies, exam sections, english language testing system, non-native english speakers

## Abstract

Non-native English-speaking physicians often encounter challenges when pursuing job and education opportunities in English-speaking countries. This article focuses on the International English Language Testing System (IELTS) exam, which is a requirement for applying to work, study, or obtain a visa in native English-speaking developed countries. The article details how the author, who is a physician from India, employed strategies that resulted in an overall band score of 8, consisting of 8.5 in listening, 9 in reading, 7.5 in writing, and 7.5 in speaking. The author had a moderate level of English proficiency before the 31-day preparation period, which involved one hour of daily practice and four-hour intensive practice for three days. To prepare, the physician used various resources, including Cambridge IELTS 15 and 16 books, two free practice tests from the British Council, a free 14-day course, and two full practice tests from Macquarie University. The author employed diverse strategies to improve their listening skills, such as listening to English content without subtitles, practicing with IELTS-like tests, and listening to tests at faster speeds. To overcome reading challenges, the author completed three reading passages during practice, read medical journals and books, and familiarized themselves with medical terminologies. For the writing section, the author practiced different question types and used language-checking software for feedback. Lastly, for the speaking section, the author practiced various topics, familiarized themselves with the exam format, and recorded themselves to identify and correct errors. This success story demonstrates how non-native English speakers can use a combination of resources and strategies to enhance their skills in all four sections of the IELTS exam at a nominal fee.

## Editorial

Introduction

Medical physicians from non-native English-speaking countries frequently seek job and educational opportunities in developed English-speaking nations, such as the United Kingdom (UK), the United States (US), Canada, Australia, and New Zealand [1]. In the former countries, medical education places greater emphasis on understanding and communicating medical concepts effectively, rather than on grammatical accuracy and English pronunciation. Consequently, this presents a significant challenge in situations where the latter factors play a critical role. For physicians seeking to immigrate to developed nations, the International English Language Testing System (IELTS) exam can potentially serve as a major hurdle [2].

The IELTS exam

There are two types of IELTS exams: Academic IELTS and General Training IELTS. The Academic IELTS is designed for those who want to pursue higher education in an English-speaking country. It assesses a candidate's ability to comprehend and use complex academic language. The General Training IELTS, on the other hand, is intended for those who want to work in an English-speaking environment, immigrate to an English-speaking country or pursue non-academic training. It assesses a candidate's ability to communicate effectively in daily life situations. Both exams have four sections: listening, reading, writing, and speaking, but the reading and writing sections differ in content and difficulty level, with the Academic IELTS being more academically-oriented and the General Training IELTS being more practical in nature. Physicians might be unable to find enough time to adequately prepare for this exam [3]. Having recently (July 2022) achieved an impressive overall band score of 8 (with a listening score of 8.5, reading score of 9, writing score of 7.5, and speaking score of 7.5) on the IELTS Academic exam, the author, a physician from India, is eager to share their experience and strategies with other non-native English-speaking physicians hoping to achieve similar success on their first attempt.

Author's baseline English proficiency before preparing for the IELTS exam

The author's educational background from school to MBBS and MD was conducted entirely in English, indicating a strong foundation in the language. However, the fact that the author is only accustomed to conversing in English for about 40% of their interactions implies limited practice in speaking and listening skills. The author also watched English movies and shows regularly, albeit with occasional reliance on subtitles due to difficulty in comprehension. This suggests potential gaps in the author's understanding of advanced vocabulary and colloquial expressions. In general, the author's English proficiency level prior to taking the IELTS exam could be described as moderate, with opportunities for improvement in specific areas.

Exam preparation strategies

The author's preparation involved devoting 31 days to the exam, with 28 days of one-hour daily practice and three days of four-hour intensive practice. The preparation materials used were minimal but included the latest Cambridge IELTS 15 and 16 books, each containing four complete test papers. In addition, the author took advantage of the two free practice tests offered by the British Council on their website. Notably, the IELTS exam is now conducted in India by the International Development Program (IDP) of Australian Universities and Colleges, and the British Council no longer offers the exam. To further augment their preparation, the author used the free 14-day course and two full practice tests provided by Macquarie University, which were offered as a complementary service with the exam registration by IDP. These resources proved to be immensely valuable, especially as the practice tests were considerably more challenging than the actual exam. As a result, they provided the author with a solid foundation for success.

Listening section

The listening section of the IELTS exam is notoriously challenging, especially for non-native English speakers. It consists of four parts, with the third section being particularly difficult as it includes lengthy multiple-choice questions. The author found this section particularly challenging as it required them to multitask by listening, reading, and writing at the same time. To overcome this challenge, the author adopted various strategies to improve their listening skills. Firstly, they regularly listened to English-language content, such as music, television series, and movies, without the aid of subtitles. This helped them to train their ear to recognize different accents, intonations, and pronunciation styles. In addition, the author found practice listening tests on the internet that closely resembled the questions found on the IELTS exam. By practicing with these tests, the author was able to familiarize themselves with the format and structure of the listening section. They even listened to the tests at 1.25x speed to build their skills for the 1x speed required during the actual exam. By utilizing these resources, the author was able to overcome the challenges of the listening section and achieve a high score in this component of the exam. This serves as an example of how non-native English speakers can effectively prepare for the IELTS exam by using a variety of resources and strategies to improve their skills in all four sections of the test.

Reading section

The reading section of the IELTS academic exam is a formidable task for many non-native English speakers, mainly due to the lengthy passages that are provided [3]. The author's success in achieving a perfect band score of 9 for this section can be attributed to their medical background, which allowed them to understand and answer questions related to the reading passages more efficiently. To prepare for this section, the author established a rigorous practice routine that involved completing three reading passages in succession during hour-long practice sessions. In addition, the author frequently read medical journals and books to improve their reading comprehension skills, which ultimately aided in quickly grasping the content of each passage within a shorter timeframe. These techniques, combined with the author's medical expertise, were instrumental in their ability to achieve a top score in the reading section of the exam.

Writing section

The writing section of the IELTS exam is notorious for its lower scores, but the author employed various strategies to achieve a high score in this section [4]. Firstly, they studied standard answers from practice tests to understand the appropriate format and language structure. Additionally, the author noticed that Native-English speakers tend to use a less repetitive and more sophisticated tone in their writing, demonstrating their strong lexical resources, coherence, and cohesion. To improve their writing skills, the author used language-checking software to proofread their writing and identify areas for improvement. However, it is important to note that while language-checking software can identify grammatical errors and spelling mistakes, it may not necessarily improve the overall quality of the writing, such as coherence and cohesion. Therefore, it is essential to supplement the use of language-checking software with practice and feedback from a qualified instructor or tutor to achieve a higher band score in the writing section of the IELTS exam.

Speaking section

Non-native English speakers often find the speaking section of the IELTS exam challenging due to a lack of regular practice. To prepare for this section, the author employed several strategies. Firstly, they engaged in daily communication with peers and family members in English throughout the preparation period, which helped them improve their fluency and confidence in speaking. Additionally, the author familiarized themselves with the commonly asked questions and preparation strategies for the speaking section by utilizing practice tests. By practicing with these tests, the author was able to improve their ability to respond to a variety of topics in a structured and organized manner. Moreover, the author watched videos of high-scoring candidates with a band score of 9 in the speaking section to gain insights into the necessary improvements required for a higher score. By observing the performance of these candidates, the author was able to understand the importance of enunciation, fluency, and pronunciation in achieving a high band score. Additionally, the author was able to identify areas of improvement, such as proper usage of grammar and vocabulary, which helped them enhance their performance in the speaking section.

Limitations of the preparation materials used by the author

Firstly, the author relied on a limited number of resources, mainly the Cambridge IELTS books and practice tests provided by the British Council and Macquarie University. While these resources were beneficial, there are other preparation materials available that may offer a wider range of content and practice materials. Secondly, the author's preparation period of 31 days may not be sufficient for everyone. Some individuals may require a longer period of preparation, especially if they have limited exposure to English or if they struggle with specific components of the exam. Finally, the author's reliance on language-checking software to proofread their writing may not be an effective strategy for everyone. While language-checking software can identify grammatical errors and spelling mistakes, it may not necessarily help improve the coherence and cohesion of the writing or the author's ability to express their ideas clearly and effectively.

Suggested alternative strategies

Alternatives to the strategies used by the author include utilizing a wider range of preparation materials, such as online courses, books, and private tutors, to gain a more comprehensive understanding of the exam content and structure. Additionally, individuals could consider extending their preparation period and focusing on specific components of the exam that they struggle with. For the speaking section, regular practice with a language exchange partner or tutor could be a beneficial strategy to improve communication skills. Furthermore, seeking feedback from a qualified IELTS instructor on practice tests and writing samples can provide valuable insights into areas that require improvement.

Conclusion

In conclusion, the author's successful preparation for the IELTS exam highlights the importance of diligent preparation and utilizing various resources, even in the face of time constraints. This experience could serve as a source of inspiration for non-native English-speaking physicians residing in developing countries who aspire to work in English-speaking countries. Passing language exams like IELTS is essential for such individuals as it demonstrates their proficiency in the English language and ensures that they can effectively communicate with patients, colleagues, and other stakeholders in the healthcare system. It is also important to note that passing these exams not only benefits the individual but also enhances the quality of healthcare delivery and patient outcomes in the host country. Therefore, it is crucial for healthcare organizations and governments to recognize the significance of language proficiency in healthcare settings and provide adequate support to non-native English-speaking physicians to help them succeed in language exams like IELTS.
